# Why they eat, what they eat: patterns of wild edible plants consumption in a tribal area of Western Himalaya

**DOI:** 10.1186/s13002-017-0198-z

**Published:** 2017-12-12

**Authors:** Deepika Thakur, Alpy Sharma, Sanjay Kr. Uniyal

**Affiliations:** High Altitude Biology Division, CSIR-Institute of Himalayan Bioresource Technology, Palampur, HP 176061 India

**Keywords:** Edible, Himalaya, Modernization, Plants, Sociocultural

## Abstract

**Background:**

From time immemorial, wild plants have been used for edible purposes. They still continue to be a major source of nutrition for tribal people. However, unfortunately, their use is now declining. This has implications in food security, narrowing genetic base, and future leads. The present study was, therefore, carried out in Chhota Bhangal region of Western Himalaya to analyze uses of wild edible plants (WEP) and the motivations behind their use or abandonment.

**Methods:**

Field surveys were conducted to the study area from January 2016 to March 2017. Household surveys, group discussions, free listing, and structured questionnaires were used to elicit information on WEP. WEP use was categorized into six categories (vegetables, fruits, chutney, flavoring food, raw food, and local brew). Trends of use (continuing, decreasing, increasing, and not used) and motivations (environmental, economic, sociocultural, agriculture and land use practices, and human-wildlife conflict) behind their use were analyzed.

**Results:**

Fifty plant species were used by the local people for edible purposes under six WEP categories. Mean and median of WEP used per respondent was 22.3 and 21, respectively. Highest number of these were used as vegetable (mean 8.9) while lowest were used as brew (mean 0.4). Out of the 50 WEP used, 20 were prioritized for motivation analyses. Though plant use is still maintained in the area, changes are evident. Almost 50% of the respondents revealed that they still continue the use of WEP while 36% reported trends of declining use as compared to 5–10 years back. Close to 10% respondents have stopped consuming WEP now and ~ 3% reported an increase in the use of WEP. Among the WEP categories, use of chutney showed an increasing trend. Sociocultural motivations were found to play a prime role, both, in limiting and promoting WEP use. Taste and aroma were the major sociocultural reasons behind using WEP while modernization and changing lifestyle were the main reasons behind declining use of WEP.

**Conclusions:**

The study concludes that though use of WEP is still maintained in the area, changes in consumption trends are evident. Sociocultural motivations guided use of WEP in the area.

## Background

Wild edible plants (WEP) represent species that are collected from the surrounding ecosystems for human consumption but are not cultivated [1]. Surrounding ecosystems include forests, pastures, and fields. FAO defines them as “plants that grow spontaneously in self maintaining populations in natural or semi-natural ecosystems and can exist independently of direct human actions” [2]. Thus, WEP are locally available, low input options for nutrition. Prior to coming up of agriculture, some 10,000 years ago, they formed a prime component of human food [3]. Throughout history, WEP have enabled humans to tide over times of wars and natural calamities [4, 5]. Thus, uses of WEP have been widely studied, both, in developed and developing countries [6–8]. It has been revealed that the number and frequency of species used varies with culture and location [9–11]. At the individual country level, 300–800 different species have been reported to be used for edible purposes [12, 13]. Another study reports that humans have used more than 7000 WEP during some stage in their history [14]. Still today, WEP complement diet of 1 million people of the world [15] and continue to be a major source of food for tribal and rural communities [16, 17]. Their importance in poverty reduction, ensuring food security, agricultural diversification, income generation, and nutrition has been specially emphasized [18–20]. It is now being argued that WEP are a rich source of vitamins and nutrients [5, 21–24] and can significantly contribute towards alleviating malnutrition [15].

On the one hand, importance of WEP is being recognized globally, on the other, a decline in their consumption as well as the knowledge associated with them is evident [25–27]. Developmental activities, socio-cultural transformations, environmental changes, lack of interest among young generation, and declining resources are cited to be the major reasons for this [21, 28–31]. Therefore, studies on WEP consumption are contemporary areas of research [32]. Such studies, especially in interior areas where dependency on natural resources is still very high and at the same time they are undergoing rapid transformations, have been emphasized [33–36].

Recognizing this, the present study was carried out in Chhota Bhangal—an interior tribal area in the Himalayan state of Himachal Pradesh. The objectives framed for the study include 1: documentation of WEP consumed in the area, and 2: identification of motivations and trends associated with their consumption.

## Methods

### Study area

The study area lies in the lap of Dhauladhar Mountain range at co-ordinates 32^°^04′32.83″ N and 76^°^51′30.45″ E in the West Himalayan state of Himachal Pradesh. Owing to its location, Chhota Bhangal receives heavy rainfall from July to September with annual rainfall close to 1500 mm. Winters are chilly, with January being the coldest month and often reporting sub-zero temperatures. Summers are usually pleasant with maximum temperature going up to 34 °C in the month of June [37]. Geologically, quartzite rocks of Saluni formation characterize the area while the soils are fertile loam to clayey loam. Uhl and Lambadug rivulets drain the area [38]. Oaks and conifer dominate the forests with birch and rhododendron forming the tree line. The area is rich in medicinal plants that are heavily traded from the region [39].

The residents of the area (referred as *Bhangalis*) are mainly agropastoralists and depend on the surrounding resources for livelihood including plants for edible purposes. Their knowledge on plants is exhibited in their local sayings and uses [40]. Natural landscape, trout farms, and adventure tourism such as paragliding are transforming the place into an important tourist destination. The 2015 world paragliding championship took place in the vicinity of the study area (http://www.pwca.org/node/24227). Therefore, the area is undergoing many developmental activities that have resulted in the movement of heavy machinery and coming up of roads [37, 38]. This has resulted in socio-economic changes and modernization in the area.

### Surveys

The work involved field surveys, interactions with *Bhangalis*, recording of data, analyses, and interpretation of the collated information. Field surveys to the study area were conducted from January 2016 to March 2017. In the initial reconnaissance surveys seven villages were identified for intensive interviews and fieldwork (Table 1). These villages are representative of the area and are located on both the banks of river Lambadug. These were selected following our earlier work in the area [39–41]. Door-to-door surveys in these villages were conducted and information on age, gender, literacy, and use of WEP was collected using structured interviews [42]. Besides, focus group discussions were also held in each village. This involved free listing of WEP and detailed notes on their methods of preparation [43]. For this, prior informed consent was taken from the people and they were informed about the purpose and nature of the study. An oral agreement to participate in the study was received from them.

**Table 1:** General profile of the village

Serial no.	Name of village	Latitude	Longitude	Altitude (meters)
1.	Termehr	32°04′28.606″	76°51′19.858″	2100
2.	Swad	32°05′09.307″	76°50′58.927″	2295
3.	Bhujling	32°06′03.73″	76°51′14.880″	2180
4.	Punag	32°05′35.753″	76°51′20.954″	2230
5.	Andarli Malahn	32°04′24.762″	76°52′01.67″	2200
6.	Napotha	32°03′58.608″	76°51′41.750″	2120
7.	Judhar	32° 04′42.06″	76° 50′50.001″	2450

Based on household surveys (*n* = 423), an inventory of WEP used by the local people was prepared (Table 2). Based on the purpose of use, these WEP were then categorized into six categories, namely, vegetables, fruits, chutney, flavoring food, raw food, and local brew (Table 3) [27, 44]. Top three to five most referred WEP in each of the category were identified for detailed analyses and documentation [31]. Thus, 20 plant species that includes a fungus were prioritized for analyzing trends and motivations behind their use [45]. Considering that local people classify fungi as a plant, the same was analyzed along with plants. For trend analyses, 176 villagers including men and women of different ages were randomly selected [30, 31]. Personal interactions using structured questionnaires were then conducted with these identified villagers (*n* = 176). Information on consumption of WEP in the past and present times was recorded. Additionally, motivations behind using WEP and reasons for their abandonment were also noted.

**Table 2:** Wild edible plants consumed in Chhota Bhangal

S. no.	Botanical name (family, collection number)	Local name	Life form	Part used	Use	Frequency of use
1.	*Aesculus indica* Hook. (Sapindaceae, PLP 9927)	Khnor	Tree	Fruits	Fruits are ground to make a flour called “seek.” Seek is then kneaded with water to prepare dish for pregnant women.	Occasionally
2.	*Allium humile* Kunth. (Amaryllidaceae, PLP 9963)	Pangri	Herb	Leaves	Thinly chopped fresh leaves are used for flavoring food.	Rarely
3.	*Allium stracheyi* Baker (Amaryllidaceae, PLP 9964)	Van lahsun	Herb	Leaves	Finely chopped fresh leaves used for infusing flavor.	Occasionally
4.	*Amaranthus paniculatus* L. (Amaranthaceae, PLP 9955)	Chaulai	Herb	Leaves	Fresh leaves are cut, fried in mustard oil, and mixed with spices.	Occasionally
5.	*Angelica glauca* Edgew. (Apiaceae, PLP 9941)	Chora	Herb	Roots	Crushed roots are used for flavoring food.	Frequently
7.	*Berberis lyceum* Royle (Berberidaceae, PLP 9937)	Kashmal	Shrub	Fruits	Ripe fruits are eaten.	Frequently
6.	*Berberis aristata* DC. (Berberidaceae, PLP 9950)	Shamle	Shrub	Fruits	Ripe fruits are eaten.	Frequently
8.	*Cannabis sativa* L. (Cannabaceae, PLP 9945)	Bhangolu	Herb	Seeds	Seeds are roasted and then consumed with sugar.	Occasionally
9.	*Capsella bursa-pastoris* Medik. (Brassicaceae, PLP 9965)	Jangli sarson	Herb	Leaves	Fresh leaves are roughly cut, boiled, and fried in mustard oil. Spices are added as per taste.	Rarely
10.	*Chenopodium album* L. (Chenopodiaceae, PLP 9931)	Bathu	Herb	Leaves and seeds	Fresh leaves are roughly cut, boiled, and fried in mustard oil. Spices are added as per taste. Seed also used for making flour.	Occasionally
11.	*Cirsium wallichii* DC. (Asteraceae, PLP 9946)	Bursa	Herb	Inflorescence	Freshly plucked inflorescence is eaten as such by children.	Rarely
12.	*Colocasia esculenta* Schott (Araceae, PLP 9961)	Kachalu	Herb	Whole plant	Young fresh leaves are chopped and boiled. They are later fried in mustard oil and spices are added to it. Tubers locally called “*kachalu*” are also boiled and then fried in mustard oil.	Frequently
13.	*Cotoneaster rotundifolius* Wall. Ex. Lindl. (Rosaceae, PLP 9962)	Riunsh	Shrub	Fruits	Ripe fruits are eaten.	Rarely
14.	*Diplazium maximum* (D.Don) C. Chr. (Dryopteridaceae, PLP 9966)	Lengadu	Fern	Young fronds (leaves)	Young and immature fronds are wiped with cloth to remove hairs and then cut into pieces and fried. While cooking spices are added. Also used for making pickles.	Almost daily (in rainy season)
15.	*Fagopyrum esculentum* Moench. (Polygonaceae, PLP 9952)	Fafra	Herb	Leaves	Fresh and young leaves are chopped, boiled and fried in mustard oil. Spices are added as per taste. In addition, the dried leaves are stored and used for making vegetable in winters.	Frequently
16.	*Foeniculum vulgare* Mill. (Apiaceae, PLP 9958)	Sounp	Herb	Seeds	Seeds are used to flavor tea.	Frequently
17.	*Fragaria nubicola* Lacaita (Rosaceae, PLP 9930)	Ban aakhre	Shrub	Fruits	Ripe fruits are eaten.	Occasionally
18.	*Impatiens glandulifera* Royle (Balsaminaceae, PLP 9936)	Tilfad	Herb	Fruits	Ripe fruits are eaten.	Occasionally
19.	*Juglans regia* L. (Juglandaceae, PLP 9959)	Khod	Tree	Fruits	Ripe fruits are eaten.	Frequently
20.	*Mentha longifolia*(L.) Huds. (Lamiaceae, PLP 9933)	Jangli pudina	Herb	Leaves	Fresh leaves are used for making chutney and also used to flavoring tea. They are ground on stone bed and spices are added to it.	Frequently
21.	*Morchella esculenta* Pers. (Morchellaceae, PLP 9967)	Guchchi	Fungus	Fruiting body	Fruiting body is chopped into pieces, boiled and then fried in mustard oil and mixed with spices.	Occasionally
22.	*Oxalis corniculata* L. (Oxalidaceae, PLP 9968)	Almori	Herb	Leaves	Fresh leaves are eaten by children.	Frequently
23.	*Oxalis latifolia* Kunth (Oxalidaceae, PLP 9940)	Malori	Herb	Leaves	Fresh leaves are eaten by children. Fresh leaves are also used for making chutney.	Frequently
24.	*Oxyria digyna* (L.) Hill (Polygonaceae, PLP 9969)	Chhoti Chukri	Herb	Leaves	Leaves are chopped into pieces and are used to make chutney.	Occasionally
25.	*Phytolacca acinosa* Roxb. (Phytolaccaceae, PLP 9953)	Jharkha	Herb	Leaves	Leaves are chopped and boiled. After boiling they are fried in mustard oil and then mixed with spices.	Occasionally
26.	*Pinus roxburghii* Sarg. (Pinaceae, PLP 9970)	Cheeltu	Tree	Seeds	Seeds are eaten raw.	Rarely
27.	*Pinus wallichiana* A.B. Jacks. (Pinaceae, PLP 9949)	Cheeltu	Tree	Seeds	Seeds are eaten raw.	Rarely
28.	*Pleurotus sp.* (Fr.) P. Kumm. (Pleurotaceae, PLP 9971)	Kyaun	Fungus	Fruiting body	The fungus is chopped into pieces, boiled and then fried in mustard oil and mixed with spices. The fungus grows abundantly on *Ulmus wallichiana.*	Frequently
29.	*Prinsepia utilis* Royle (Rosaceae, PLP 9944)	Bhekal	Shrub	Fruits	Ripe fruits are eaten.	Rarely
30.	*Prunus armeniaca* L. (Rosaceae, PLP 9938)	Shaade	Tree	Fruits	Ripe fruits and nuts are eaten.	Frequently
31.	*Prunus cornuta* Steud. (Rosaceae, PLP 9929)	Jamnu	Tree	Fruits	Ripe fruits are eaten.	Frequently
32.	*Prunus persica* Batsch (Rosaceae, PLP 9960)	Aaru	Tree	Fruits	Ripe fruits are eaten.	Occasionally
33.	*Pyrus pashia* Buch.-Ham. Ex D.Don. (Rosaceae, PLP 9932)	Shegal	Tree	Fruits	Ripe fruits are eaten.	Occasionally
34.	*Rheum australe* D. Don (Polygonaceae, PLP 9972)	Chambu	Herb	Leaves	For making vegetable, leaves are chopped into pieces, boiled and fried in mustard oil and mixed with spices.	Occasionally
35.	*Rhododendron arboreum* Sm. (Ericaceae, PLP 9954)	Braah	Tree	Flower	Flowers are used to make chutney with mint and also dried under sun for use in future.	Frequently
36.	*Rosa canina* L. (Rosaceae, PLP 9951)	Jangli Gulaab	Shrub	Fruits	Ripe fruits are eaten.	Occasionally
37.	*Rubus ellipticus* Sm. (Rosaceae, PLP 9973)	Aakhre	Shrub	Fruits	Ripe fruits are eaten.	Frequently
38.	*Rubus foliolosus* D. Don (Rosaceae, PLP 9943)	Aakhre	Shrub	Fruits	Ripe fruits are eaten.	Frequently
39.	*Rumex dentatus* L. (Polygonaceae, PLP 9957)	Milu	Herb	Leaves	Fresh leaves are eaten by children.	Frequently
40.	*Rumex hastatus* D. Don (Polygonaceae, PLP 9939)	Jhemlu	Herb	Aerial part	Aerial parts are eaten raw and also used for making chutney with mint.	Frequently
41.	*Selinum tenuifolium* Wall. ex C.B. Clarke. (Apiaceae, PLP 9956)	Matoshal	Herb	Roots	Locally called dheli, roots are used in making local brew.	Rarely
42.	*Silene vulgaris* Garcke (Caryophyllaceae, PLP 9974)	Bibdughas	Herb	Leaves	Fresh leaves are chopped and boiled. After boiling, fried in mustard oil and mixed with spices.	Occasionally
43.	*Sonchus asper* Hill (Asteraceae, PLP 9934)	Dudala	Herb	Leaves	Fresh leaves are chopped and boiled. After boiling, fried in mustard oil and mixed with spices.	Occasionally
44.	*Stellaria media* Vill. (Caryophyllaceae, PLP 9942)	Khokhua	Herb	Aerial parts	Aerial parts are chopped, boiled and fried in mustard oil. Spices are added while cooking.	Frequently
45.	*Taraxacum officinale* Weber ex F.H. Wigg. (Asteraceae, PLP 9935)	Shershi	Herb	Leaves	Fresh leaves are chopped into pieces and boiled. After boiling they are fried in mustard oil and mixed with spices.	Frequently
46.	*Taxus baccata subsp. wallichiana* (Zucc.) Pilg. (Taxaceae, PLP 9948)	Rakhal	Tree	Bark and leaves	Bark and leaves of Rakhal are used for flavoring tea.	Occasionally
47.	*Thymus linearis* Benth. (Lamiaceae, PLP 9975)	Van Ajwain	Herb	Seeds	Seeds are used for flavoring tea.	Frequently
48.	*Urtica dioica* L. (Urticaceae, PLP 9928)	Kushak	Herb	Young leaves	Young and fresh leaves are chopped into pieces, boiled and then fried in mustard oil and mixed with spices.	Rarely
49.	*Viola pilosa* Blume (Violaceae, PLP 9947)	Banaksha	Herb	Flower and leaves	Flower and leaves of banaksha are used for flavoring tea.	Occasionally
50.	*Zanthoxylum armatum* DC. (Rutaceae, PLP 9976)	Tirmir	Shrub	Fruits	Ripe fruits are eaten.	Rarely

**Table 3:** Wild edible plant categories and their characteristics

WEP category	Characteristics
Vegetables	Species that are cooked as food
Fruits	Species of which fresh/dry fruits are consumed without cooking
Chutney	Species ground with salt and spices for preparing sauce
Flavoring food	Species used for seasoning and infusing aroma
Raw food	Species in which fresh plant part, other than fruit, is eaten raw such as salad
Local brew	Species used to prepare liquor

Data so collected were analyzed for species richness, taxonomic diversity, and plant part used for edible purposes. Analyses of trends of WEP use were also done. These trends/patterns were categorized into continuing use, declining use, increasing use, and not used (Table 4). The motivations/reasons behind these trends were then identified. These motivations encompass explanations pertaining to environment, economy, sociocultural, agriculture and land use changes, and human-wildlife conflict (Table 5). Voucher specimens were collected and have been deposited in the herbarium of CSIR-Institute of Himalayan Bioresource Technology, Palampur (Acronym PLP).

**Table 4:** Categorization of use trends

Trend	Characteristics
Continuing use	Species that were consumed earlier and are still consumed in similar proportions
Declining use	Species that were consumed in higher amounts earlier (5 years) but now less used
Increasing use	Species that were consumed in lesser amount in the past but now more used
Not used	Species that were used earlier but are not used now

**Table 5:** Characteristics of different motivation categories

Motivation category	Characteristics
Environmental	Explanations related to factors such as climate, abundance and scarcity
Economic	Explanations pertaining to factors such as price, market value, and commercial availability
Sociocultural	Explanations related to taste, aroma, flavor, health, ritual, and interest
Agriculture and land use practices	Explanations related to changing agriculture and land use such as cultivation of cash crops, etc.
Human-wildlife conflict	Explanations related to crop depredation by wild animals, attacks by wild animals on humans, etc.

## Results

### WEP diversity

**Fig. 1:**
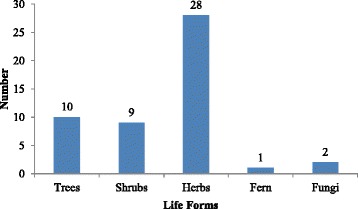
Life form categorization of the species used in Chhota Bhangal

**Fig. 2:**
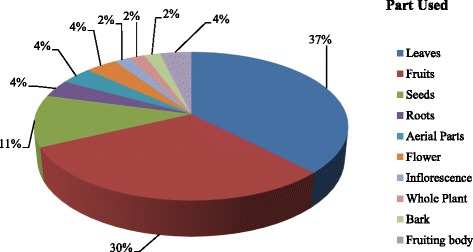
Statistics of different plant parts used

The *Bhangalis* reported use of 50 plant species belonging to 42 genera falling under 28 families as WEP. Majority of these belong to family Rosaceae (10 spp.) followed by Polygonaceae (5 spp.), Apiaceae, and Asteraceae (3 spp. each). Oxalidaceae, Lamiaceae, Pinaceae, Berberidaceae, Caryophyllaceae, and Amaryllidaceae were represented by two species each. Remaining families (*n* = 17) had one species each (Table 2). Herbs dominated the WEP that were consumed (*n* = 28, 56%) followed by trees (*n* = 10, 20%) and shrubs (*n* = 9, 18%). One species of fern (2%) and two species of fungi (4%) were also eaten (Fig. 1). Among the plant parts used, mostly leaves were used for different preparations (*n* = 20 species, 37%) followed by fruits (*n* = 16 species, 30%), seeds (*n* = 6 species, 11%), and fruiting bodies (*n* = 2 species, 4%). Roots, aerial parts, and flowers of two species each (4%) were used. Inflorescence, whole plant and bark were the least used parts (*n* = 1 species each, 2%) (Fig. 2).

Overall, mean number of species listed and used per respondent was 23.7 (median − 21) and 22.3 (median − 21), respectively. Most of these were used as vegetable (mean − 8.9, median − 9) followed by fruits (mean − 6.3, median − 6). An average of 3.2 species (median − 3) were used as chutney whereas the mean number of species used as flavoring foods was 2.2 (median − 2). The average number of species per respondent in case of raw food and local brew was 1.4 (median − 1) and 0.4, respectively.

### Trends in WEP consumption

As mentioned in methods, of the total 50 WEP, 20 species were prioritized for trend analyses. These include four used as vegetables, five as fruits, and four each as chutney and flavoring food, three as raw food, and one species as local brew (Table 6). *Oxalis latifolia* was used both as raw food and chutney. Species falling under the head fruits and raw food were consumed as such by the people. These include *Berberis aristata*, *Juglans regia*, *Oxalis corniculata*, *Oxalis latifolia*, *Prunus armeniaca*, *Prunus cornuta*, *Rubus ellipticus*, and *Rumex dentatus*. Plants collected for other purposes were consumed after processing. The study revealed that more than 50% of the respondents had ever consumed the prioritized WEP in the area during their lifetime.

**Table 6:** Prioritized plant species in different WEP categories

Categories	Botanical name	Local name
Vegetables	*Fagopyrum esculentum*	Fafra
	*Diplazium maximum*	Lengadu
	*Pleurotus sp.* Wild mushroom on *Ulmus wallichiana*	Kyaun
	*Colocasia esculenta*	Kachalu
Fruits	*Prunus armeniaca*	Shaade
	*Juglans regia*	Khod
	*Rubus ellipticus*	Aakhre
	*Berberis aristata*	Shamle
	*Prunus cornuta*	Jamnu
Chutney	*Rhododendron arboreum*	Braah
	*Mentha longifolia*	Jangli pudina
	*Rumex hastatus*	Jhemlu
	*Oxalis latifolia*	Malori
Flavoring food	*Angelica glauca*	Chora
	*Viola pilosa*	Banaksha
	*Foeniculum vulgare*	Sounp
	*Thymus linearis*	Van ajwain
Raw food	*Oxalis corniculata*	Almori
	*Rumex dentatus*	Milu
	*Oxalis latifolia*	Malori
Local brew	*Selinum tenuifolium*	Matoshal

Among the six WEP categories, most of the respondents (68.75%) favored continuing use of chutney and only 5.68% respondents favored continuing use of WEP for local brewing. With respect to vegetables, 64.77% of the respondents reported a continuing use trend while 35.23% revealed its declining use (Table 7). On comparing continuing and declining use, highest number of respondents in all the WEP categories were for continuing use barring flavoring food and local brew, where the percentage of respondents was highest for declining use, i.e., 54.55 and 50.57%, respectively (Table 7). In case of chutney, an increasing use trend was observed. Close to 23 % of the respondents who were not consuming it earlier are now consuming it. This was the only WEP category in which an increasing use trend was recorded (Table 7). It was found that few respondents had not used the prioritized plant species during the past 5 years. This includes 1.14% of the respondents for chutney category, 7.95% for flavoring food, 9.66% for raw food, and 43.75% for the local brew.

**Table 7:** Trends in use (%) across different WEP categories

WEP category	Trends			
	Continuing use	Declining use	Increasing use	Not used
Vegetable	64.77	35.23	–	–
Fruits	68.18	31.82	–	–
Chutney	68.75	6.82	23.30	1.14
Flavoring food	37.50	54.55	–	7.95
Raw food	50.57	39.77	–	9.66
Local brew	5.68	50.57	–	43.75
Total	49.24 ± 10.03	36.46 ± 6.93	3.88 ± 3.88	10.42 ± 6.88

None of the respondents reported of having not used the prioritized vegetables and fruits during the past 5 years.

Overall, irrespective of the WEP category, 49.24% of respondents continue use of WEP while 10.42% reported not using them now. Declining use of WEP was reported by 36% of the respondents (Table 7).

### Motivations for WEP consumption

There were a total of 1341 responses offered by the 176 respondents for motivations that inspire or limit WEP consumption. A similar response given by two different respondents has been counted as two individual responses. As detailed in the methods, all these responses have been clubbed under five different motivation categories, namely, environmental, economy, sociocultural, agriculture and land use changes, and human-wildlife conflict.

Of the total 1341 responses, 743 (55.41%) were for motivations leading to the continuing use of WEP, while 529 (39.45%) were for their declining use (Table 8). Highest number of responses (82.55%) fall under the sociocultural motivation category and lowest under the agriculture and land use changes category. Environmental motivations accounted only for 11.48% of the total responses, while only 2.83% cited the economic motivations (Table 8).

**Table 8:** Responses under different motivation categories that guide WEP use

Motivation category	Trends for consumption							
	Continuing		Declining		Increasing		Total	
	N	%	N	%	N	%	N	%
Environmental	42	3.13	112	8.35	0	0	154	11.48
Economic	31	2.31	7	0.52	0	0	38	2.83
Sociocultural	670	49.96	368	27.44	69	5.15	1107	82.55
Agriculture and land use changes	0	0	5	0.37	0	0	5	0.37
Human-wildlife conflict	0	0	37	2.76	0	0	37	2.76
Total	743	55.41	529	39.45	69	5.15	1341	100.00

With respect to continuing, declining, and increasing use, highest responses, i.e., 670 (49.96%), 368 (27.44%) and 69 (5.15%), respectively fall under the sociocultural motivation category (Table 8). Responses under motivation categories agriculture and land use changes (0.37%), and human-wildlife conflict (2.76%) accounted only for declining use of WEP (Table 8).

### Motivation explanations

It was observed that WEP consumption was guided by 25 motivation explanations under the five motivation categories (Table 9). Sociocultural category accounted for largest number of the explanations (*n* = 17) followed by environmental (*n* = 3), economic and human-wildlife conflict (*n* = 2 each), and agriculture and land use changes (*n* = 1) (Table 9). With respect to consumption trends, out of 25 motivation explanations, 9 explanations were for continuing use, while 12 were for declining use, and the remaining for increasing use. As pointed earlier, only WEP chutney category had an increasing use trend. Here-in four explanations were given for increasing use by 41 respondents. Of the 17 explanations that represented sociocultural motivation category, 7 were for continuing use, 6 for decreasing use, and 4 for increasing use of WEP (Table 9).

**Table 9:** People’s responses and explanations that guide WEP consumption in the area

Motivation category	Motivation explanation	Trend	Example	Total	Percentage
Environmental	1. It is abundantly and easily available	Continue	They are in large quantity	42	3.13
	2. It is scare due to changing environment	Decline	They are few because of low/high rainfall	70	5.22
	3. Difficult to access	Decline	Grows at high altitude	42	3.13
Economic	4. It is free of cost	Continue	We do not pay for it	31	2.31
	5. Now not available in the local market	Decline	It is sold out of local market	7	0.52
Sociocultural	6. Lovely aroma and flavor	Continue	I love it	45	3.36
	7. Good and yummy taste	Continue	It is so tasty	490	36.54
	8. Local food	Continue	It is a food of our area	4	0.30
	9. Traditional culture	Continue	It is in our tradition	21	1.57
	10. Healthy nature	Continue	It is good for our health	43	3.21
	11. Having medicinal properties	Continue	It is good for stomach	47	3.50
	12. It is eaten for the sake of interest	Continue	I am interested in eating it	20	1.49
	13. Tasteless and bad aroma	Decline	I do not love it	5	0.37
	14. Lack of knowledge about plant	Decline	I do not have much knowledge about the plant	71	5.29
	15. Market availability	Decline	It is replaced by market goods	22	1.64
	16. Culture and lifestyle changes	Decline	Our diets have changed	222	16.55
	17. Restrictions for eating	Decline	It is unhealthy	5	0.37
	18. Large time is needed to collect it/waste of time	Decline	I do no collect it	43	3.21
	19. Good and yummy taste	Increase	It is tasty	25	1.86
	20. Culture and lifestyle changes	Increase	I am interested in eating it	39	2.91
	21. Healthy nature	Increase	It is good for health	1	0.07
	22. Having medicinal properties	Increase	It is good for stomach	4	0.30
Agriculture and land use changes	23. Changes in agricultural activities	Decline	Changed agricultural practices reduce availability of WEP	5	0.37
Human-wildlife conflict	24. Animal destruction	Decline	Monkey destroy them	32	2.39
	25. Human disturbance	Decline	Forest destruction by humans	5	0.37
Total				1341	100

In general, it was observed that out of the total responses, highest (36.54%) were related to taste and were instrumental in continuing consumption of WEP. Close to 17% of the responses were related to changing lifestyle that accounted for declining use of WEP (Table 9). Both of these represent the sociocultural motivation category. Lack of knowledge regarding the specific use of plants species (5.29%), and reduced availability of species due to changing environmental conditions (5.22%) were the other common responses behind the declining use of WEP (Table 9). Local interest and preference for taste were the two main reasons (2.91 and 1.86%, respectively) for the increasing use of WEP (Table 9). Free availability (2.31%) also had a role to play in continuing use. Changes in land use practices (0.37%), and human-wildlife conflicts such as forest degradation, attacks on humans, and crop depredation by wild animals also resulted in the declining use of WEP (2.76%) (Table 9).

## Discussion

Wild edible plants continue to satiate human diet especially in interior areas such as the Himalaya [24]. Out of the total 323 plant species reported to be edible in the entire state of Himachal Pradesh [46], almost 15% are used by the *Bhangalis*. Close to 50% of the respondents reported use of WEP. Studies among the Tibetan communities in Gansu province of China have shown use of 54 wild vascular plant species for edible purposes. The mean and median of wild edible plants used in the area was 20.8 and 21, respectively [47]. In the present study, though total number of WEP species reported was comparatively low (50), mean of WEP used was higher (22.3). On the other hand, in Poljica and Krk islands, Croatia total number of wild edible species used was higher [48]. However, mean species used per interview was low [47, 48]. It reveals that local people individually use more species in the present study area. In Imereti region of Western Georgia, 53 wild species with a mean of 10.4 species per interview have been reported to be used for edible purposes [49]. Similar results have also been presented from the Gongba Valley of China [11].

Alike other studies, vegetables formed a prime component of WEP in the area. In the present area, mean number of species used as vegetables per respondent (8.8) was higher than mean number of species used in any other WEP category. Here, alike other Himalayan regions, people dry the seasonally available vegetables for use during periods of snowfall [11]. This shows the importance of plants for sustenance and nutrition in the interior areas. A mean of 7.5 species per interview has been reported to be used as vegetables in the Gansu province of China [47], while 8.7 species of green vegetables per interview has been reported to be used in Gongba valley of China [11]. At the same time, similar mean number of species as in the present area (6.3) have been reported to be used as fruits in the Gansu province of China (6.3). In Gongba valley of China and Imereti region of Western Georgia, mean number of species used as fruits is 6.9, each [11, 47, 49].

Trends in changing WEP use patterns are evident in the area as has been reported from across the globe [8, 32, 50]. Declining use of WEP has severe implications for future prospections and leads to narrowing of genetic base [9, 30, 31]. In the present study, people reported increasing use of WEP as chutney (23.30%), more than 50% reported a declining use of WEP as flavoring food (54.55%) and also as local brew (50.57%). The reasons for this could be low populations of flavoring food species and the extra effort required for their collection. Findings from Saaremaa, Estonia show similar patterns where people would not like to go to distant places and search for WEP now [51]. In Patagonia, South America also targeted efforts for collecting wild foods has limited their gathering and use [52]. At times, regulations also have a role to play in declining use of WEP. In the present area, local alcohol brewing requires permission, otherwise it is illegal. In Catalan Pyrenees and Balearic Islands also restrictions were pointed as a reason leading to declining use of WEP [31]. Increasing use trend of chutney may be because WEP used for making chutney are often found around villages and are required in low quantity. Few workers have also reported such an increase in consumption of specialized plant species where the volume required is low [30]. In the present area, *Aesculus indica* is used as a specialized food for expecting mothers. The fruits of this plant are thoroughly washed, dried, and ground. The flour so obtained is used for making the recipe—*seek*. Use and preparation of the recipe has been provided in a separate publication by the authors [40]. Studies on WEP that require specialized processing are now receiving much attention. This has been discussed in detail for the comfrey and buttercup eaters of the Imereti region, Western Georgia [49].

**Fig. 3:**
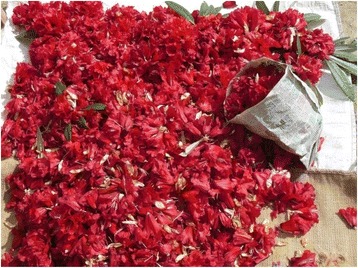
Flowers of *Rhododendron* being sold in the market for making chutney

**Fig. 4:**
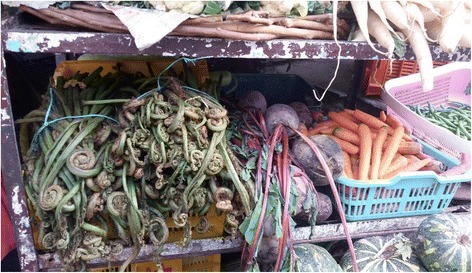
Fronds of *Diplazium* put up for sale (extreme left)

Irrespective of the WEP category, sociocultural motivations were found to play an important role in defining continuing, declining, and increasing use of WEP in the present study. Elsewhere also, studies have highlighted sociocultural factors to play a major role in WEP use [23, 25, 30, 53]. In the Catalan Pyrenees and Balearic Islands, taste was reported to be a prime motivation for continuing consumption of WEP while lifestyle changes led to abandonment of WEP consumption [31]. In Saaremaa, Estonia also use of WEP have been related to taste [51] while the disappearance of familiar taxa from surrounding areas has been linked to their declining use [51]. Similarly, while taste and aroma were the major sociocultural motivations for continuing use of WEP in the present study, changing lifestyle pattern was the prime reason for their declining use. Many studies have noted the importance of traditional culture in maintenance of WEP consumption [54, 55]. In Iberian Peninsula, despite an overall decreasing trend, uses of WEP of high cultural appreciation and recreation was found to be still maintained [30]. On the other hand, modern lifestyle and market availability of resources have limited the use of WEP [21, 28, 29, 56–58]. In some areas, collection of WEP is now seen as something that is old fashioned [59]. Interestingly, we found, people prefer to sell WEP in the market and earn hard cash rather than consume WEP themselves (Figs. 3 and 4). As reported, market forces and changes in agriculture and land use practices affect traditional lifestyle and WEP use [60, 61]. In the present area, cash crops are replacing traditional crops and thus land use changes are also responsible for declining use of WEP. Conversion of forest land and degradation of resources leads to human-wildlife conflict, which in turn also limits WEP use. In Saaremaa, Estonia, though in minor proportions, the fear of poisonous snakes and insects has limited the use of WEP [51]. This has also been noted in Catalan Pyrenees and the Balearic Islands [31], and in Rio Grande do Sul, south Brazil [62].

In the present study, close to 3% of the responses point to easy access and large population as prime reasons behind consumption of WEP. This clearly indicates that people do not like to wander into the interiors for searching WEP. They would rather prefer using plants that are available in their vicinity. It has been pointed out that free and easy availability of resources motivates its use by the local inhabitants [59]. Further, WEP with multiple utility such as associated health benefits are preferred for consumption [26, 51]. In Patagonia, changing environmental conditions are documented to have negatively affected WEP consumption [63]. Interestingly, in the present area also people cited that shortfall in rains has resulted in a decline in availability of some species (especially ferns). This consequently, has limited their use.

## Conclusion

The study concludes that though use of wild edible plants is still maintained in the area, a change in consumption trends is evident. Sociocultural motivations were found to play a prime role in, both, limiting and promoting WEP consumption. While taste and aroma were the major sociocultural reasons behind using WEP, modernization, and changing lifestyle were the main reasons behind declining use of WEP.

## Abbreviations

FAO: Food and Agriculture Organization
NMHS: National Mission on Himalayan Studies
PLP: Palampur
WEP: Wild Edible Plants
